# Cross-Sectional Study to Determine the Prevalence of Hepatitis B and C Virus Infection in High Risk Groups in the Northeast Region of Brazil

**DOI:** 10.3390/ijerph14070793

**Published:** 2017-07-17

**Authors:** Jakeline Ribeiro Barbosa, Cristianne Sousa Bezerra, Filipe Anibal Carvalho-Costa, Carolina Pimentel de Azevedo, Geane Lopes Flores, Jeová Keny Baima Colares, Danielle Malta Lima, Elisabeth Lampe, Lívia Melo Villar

**Affiliations:** 1 Viral Hepatitis Laboratory, Oswaldo Cruz Institute, Oswaldo Cruz Foundation (FIOCRUZ), Rio de Janeiro 21040-900, Brazil; jakelinebarbosa@gmail.com (J.R.B.); tiannebezerra@gmail.com (C.S.B.); carolazevedo09@msn.com (C.P.d.A.); geane@ioc.fiocruz.br (G.L.F.); elampe@ioc.fiocruz.br (E.L.); 2 Postgraduate Programme in Pathology, Federal University of Ceará State, Fortaleza CE 60430-160, Brazil; kenycolares@unifor.br (J.K.B.C.); danimalta37@gmail.com (D.M.L.); 3 Laboratory of Epidemiology and Molecular Systematics, Oswaldo Cruz Institute, Fiocruz, Rio de Janeiro 21040-900, Brazil; guaratiba@ioc.fiocruz.br; 4 Postgraduate Programme in Medical Sciences, University of Fortaleza, Fortaleza CE 60811-905, Brazil

**Keywords:** prevalence, hepatitis B virus, hepatitis C virus, human immunodeficiency virus 1, haemodialysis, coagulopathy

## Abstract

*Background*: HBV (Hepatitis B Virus) and HCV (Hepatitis C Virus) infections are more prevalent in vulnerable populations than the general population. The objective of this study was to investigate the prevalence of HBV and HCV infection in HIV-positive patients (GI), chronic renal failure (CRF) patients (GII) and coagulation disorder individuals (GIII). *Methods*: A cross-sectional study was conducted from June 2014 to March 2015. Serum samples were tested for markers of hepatitis B and C by enzyme-linked immunosorbent assay (ELISA). Sociodemographic, epidemiological, clinical and laboratory data and accompanying statistical analyses were performed using Epi Info™ 7. *Results*: A total of 348 individuals were recruited, i.e., 154 HIV-positive, 143 CRF and 51 coagulopathy patients. Among them, more than 66% were men, and the predominant age group was 26–35 years in GI and 56–65 years in GIII. Most patients had more than 8 years of education (66.2% in GI, 60.6% in GIII and 46.1% in GII), with a family income between 100–400 dollars in more than 48% of patients. The prevalence of the HBsAg marker was 3.9%, 7% and 3.9%, total anti-HBc was 28.6%, 55.9% and 31.4%, and anti-HCV was 1.3%, 12.6% and 47% for GI, GII and GIII, respectively. However, the prevalence of anti-HBs was greater than 70% in all groups. *Conclusions*: This study shows a high prevalence of HBV and HCV among specific groups compared to the general population. Factors such as age, income, number of sexual partners, sexually transmitted disease burden, blood transfusion history or blood products and blood transfusions before 1994 were associated with a higher prevalence for these infections.

## 1. Introduction

Hepatitis B Virus (HBV) and Hepatitis C Virus (HCV) can cause acute and chronic liver disease, which can progress to cirrhosis, liver failure and/or hepatocellular carcinoma [1,2]. An estimated 240 million people are chronically infected with hepatitis B virus, which is defined as testing positive for hepatitis B surface antigen for at least 6 months. Of these cases, more than 686,000 people die every year due to hepatitis B complications, such as cirrhosis and liver cancer [3]. In Brazil, the prevalence of antibodies against HBc (anti-HBc) ranged from 8.1% to 10.3% in the Federal District and the Central-West region, while the prevalence of HBsAg antigen was 0.19% in the Northeast, 0.47% in the Central-West and 0.60% in the Federal District [4].

It is estimated that approximately 130–150 million people worldwide have chronic hepatitis C infection and that approximately 700,000 people die each year from liver disease related to hepatitis C [3]. In Brazil, the prevalence of Hepatitis C antibodies (anti-HCV) was 1.38% in the state capitals of the five macro-regions and the Federal District, and it varied from 0.68% in the Northeast to 2.10% in the North region of the country [5].

HBV and HCV infections are prevalent worldwide, especially in the most vulnerable populations, such as patients infected with human immunodeficiency virus (HIV), patients with chronic renal failure (CRF) that are on haemodialysis and patients with coagulation disorders compared to the general population [6,7,8,9,10,11,12,13,14].

HBV and HCV prevalence is high among individuals infected with HIV due to the shared routes of transmission of infection, such as injecting drug use, blood transfusion and sexual intercourse [11,13,15]. In Brazil, the prevalence of HIV/HBV co-infection ranged from 2.8% to 10.3%, while HIV/HCV co-infection ranged from 4.6% to 6.4% [14,16,17]. The high prevalence of co-infected individuals varied according to the specific region of the country [18].

CRF patients are considered high-risk for acquiring HBV and HCV infections because parenteral exposure during the dialysis treatment is a major route of transmission. These infections negatively impact the survival of the haemodialysis patients and those undergoing renal transplant. In Brazil, the prevalence of anti-HBc and HBsAg were 34.1% and 15.4%, respectively, while the prevalence of anti-HCV ranged from 12% to 33.9% in CRF patients [7,8,9,19]. 

Hereditary coagulopathies are the result of deficiency diseases of one or more plasma clotting proteins. Among the inherited bleeding disorders, haemophilia and Von Willebrand’s Disease (VWD) are the most common. Haemophilia A and B are X-linked recessive bleeding disorders included among rare diseases and caused by mutations in the factor VIII and factor IX genes. Both factors take part in the intrinsic pathway of blood coagulation, and affected individuals have severe, moderate and mild forms of the diseases, which are defined by factor plasma levels [20,21,22]. However, VWD is caused by a decrease in or dysfunction of the protein called Von Willebrand Factor (VWF) and affects both genders. The diversity of mutations results in various clinical manifestations, e.g., platelet dysfunction associated with decreased serum factor VIII levels [21].

Treatment of coagulopathies involves the replacement of deficient clotting factors, which is administered as processed concentrates from blood donors and/or synthesized by the pharmaceutical industry. Clotting factor replacement increases the survival of coagulopathy patients. However, these patients are at an increased risk of infection with HBV and HCV because of multiple blood transfusions and frequent parenteral exposure. In Brazil, the prevalence of HBV infection ranged from 0.7% to 2.3%, while HCV infection ranged from 2.5% to 34.9% in this population [12,22,23].

Brazilian studies to determine the prevalence of HBV and HCV infection are common in the general population, mainly in the Southeast region of the country. However, few studies have been conducted including these specific populations in Northeast Brazil, such as HIV-positive individuals, patients with CRF on haemodialysis and patients with coagulation disorders [17]. Identifying the prevalence of these infections in these more vulnerable groups is critical to diagnose, treat and prevent the spread of these infections. Therefore, this study aimed to investigate the prevalence of HBV and HCV infection in HIV-positive patients, CRF patients on haemodialysis and coagulation disorders in Fortaleza-CE, Brazil.

## 2. Materials and Methods 

### 2.1. Study Design, Sampling and Population

A cross-sectional study was conducted during the period of June 2014 to March 2015. Sociodemographic data from subjects were obtained through a questionnaire, including the variables of income, risk behaviour, age, gender, marital status and education. 

Three groups included were as follows: I—HIV-infected patients (*n* = 154); II—patients with CRF on haemodialysis (*n* = 143); and III—patients with coagulopathy (haemophilia or von Willebrand disease) (*n* = 51). These groups were recruited, respectively, from a private HIV/AIDS clinic, private haemodialysis clinic and public Hematology and Hemotherapy Center of Ceará city, all of which are located in Fortaleza, Northeast region of Brazil.

The inclusion criteria for the selection of participants were as follows: individuals of both genders, aged 18 years or more and referral to one of the three health service centres involved in this study (group I, II or III). In addition, diagnostic criteria for HIV (group I) was considered, which included individuals who tested positive using the HIV 1/2 STAT-PAK^®^ kit (Chembio Diagnostic Systems Inc., Medford, NY, USA) and who may or may not have undergone antiretroviral treatment for HIV. Group II included CRF patients who were already diagnosed and undergoing haemodialysis treatment in the nephrology clinic. Group III included patients with haemophilia- (A and B) and VWD-type coagulopathies who were diagnosed by clinical and laboratory parameters at the Center of Hematology of Ceará, where patients are monitored since childhood.

### 2.2. Laboratory Analysis

Blood samples (10 mL) were collected from each subject by venipuncture, centrifuged to obtain serum and stored at −20 °C until analysis. Serum samples were tested for markers of hepatitis B and C by enzyme-linked immunosorbent assay (ELISA) using commercially available kits. All samples were examined for the presence of HBsAg (Bioelisa^®^ HBsAg, Biokit, Lliçà d’Amunt, Spain), total anti-HBc (anti-HBc Bioelisa^®^, Biokit), anti-HBs (anti-HBs Bioelisa^®^, Biokit) and anti-HCV (Murex anti-HCV version 4.0^®^, DiaSorin, Saluggia, Italy) according to the manufacturer’s instructions. Reactive and indeterminate samples were retested in duplicate and indeterminate results were excluded. Serological tests were performed at the Viral Hepatitis Laboratory of the Oswaldo Cruz Institute—FIOCRUZ.

### 2.3. Data Analysis

Sociodemographic, epidemiological, clinical and laboratory data were entered Microsoft Access^®^ database (Microsoft, Redmond, WA, USA). Statistical analyses were performed using Epi Info™ 7 (Epi Info Software, CDC, Atlanta, GA, USA). Prevalence rates in distinct groups were compared with Fisher’s exact test. Results with *p* values (two-tailed) < 0.05 were considered statistically significant.

### 2.4. Ethical Aspects

The Ethics Committee of FIOCRUZ approved this study under the numbers 34049514.7.3006.5258 and 34049514.7.3009.5051. All patients included in the study provided informed consent and study protocols were followed according to the ethical guidelines of the 1975 Declaration of Helsinki.

## 3. Results

A total of 348 patients were recruited for this study, which included 154 HIV-infected patients, 143 patients with CRF on haemodialysis and 51 patients with coagulopathies, with at least 68% of these patients being men. More than 55% were 26–35 years of age in the HIV group (GI) and coagulopathy group (GIII), while the predominant age was 56–65 years in haemodialysis group (GII) (Table 1).

Regarding marital status, 70.8% of group I and 66.7% of group III were single. However, approximately 50% of group II subjects were married or had a stable union. The education of the participants was greater than 8 years in 66.2% of group I, 60.6% of group III and 46.1% of group II. Of which, more than 48% of participants had a monthly family per capita income between 100 and 400 dollars.

As shown in Table 2, the prevalence of the HBV surface antigen (HBsAg) was 3.9%, 7% and 3.9% for groups I, II and III, respectively. While the prevalence of total anti-HBc marker was 28.6%, 55.9% and 31.4% in GI, GII and GIII, respectively, anti-HBs prevalence was greater than 70% in all groups. The presence of total anti-HBc and anti-HBs, which indicated immunity after HBV infection, was 21.4% (33/154) in group I, 38.5% (55/143) in group II and 29.4% (15/51) in group III.

The prevalence of HCV antibody (anti-HCV) was 1.3% (2/154), 12.6% (18/143) and 47% (24/51) in HIV-positive, CRF-haemodialysis and coagulopathy groups, respectively. 

Table 3 shows the prevalence of total anti-HBc antibody in relation to the risk factors for each group, with males being the most prevalent, i.e., 31.1% (41/131) of group I, 56.1% (55/98) of group II 32% (16/50) of group III. The prevalence of total anti-HBc in the respective groups was 36.9% (17/46) in the age range of 36–45 years, 69.4% (25/36) in the age range of 56–65 years, and 50% (6/12) in the range of 36–45 years. There was a statistically significant difference between the frequency of this marker in the different age groups of group II (*p* = 0.013) and group III (*p* = 0.025).

Regarding the monthly family per capita income, the highest prevalence of anti-HBc was among individuals without income in group I (50%, 4/8), among individuals with an income higher than $400 in group II (69.4%, 25/36) and among those without income in group III (75%, 3/4, *p* = 0.059).

Regarding sexual behaviour, men who had sex with men (MSM) had a higher prevalence of total anti-HBc (34.5%, 30/87) in group I, while the highest prevalence of anti-HBc in groups II and III (55.9%, 52/93; 32.6%, 16/49) was among non-MSM subjects. Regarding the number of sexual partners in the previous year, the prevalence of anti-HBc in group I was higher among those who had more than 10 partners (*p* = 0.003). In group II, anti-HBc was more prevalent among those who had six partners or more. While in group III, it was higher among those who had only one partner in the last twelve months (45%, 9/20). There was no statistically significant correlation between the use of a condom and the frequency of anti-HBc in the groups.

The prevalence of anti-HBc was higher for those individuals with coagulopathy (GIII) who had already undergone blood or plasma transfusion (*p* = 0.018) and for blood transfusions performed before 1994 (*p* = 0.076), since testing for anti-HCV became a mandatory component of serological screening conducted at Brazilian blood banks after November 1993.

Table 4 shows the prevalence of anti-HCV according to the socioeconomic and behavioural characteristics of each group; there was a prevalence of 4.3% (1/23) among females and 3.2% (1/131) among males in group II (15.3%, 15/98) and group III (48%, 24/50). The highest age range for anti-HCV was 46–55 years (5.2%, 1/19), 56–65 years (22.2%, 8/36) and 36–45 years (91.6%, 11/12) in GI, GII, and GIII, respectively. Married/stable union individuals and those with 1–8 years of education had a higher prevalence of anti-HCV.

Participants in this study with a monthly family per capita income of less than 100 dollars had a higher prevalence of anti-HCV in group I (6.4%, 1/16) and group II (14.3%, 3/21), while group III had a prevalence of 56% (14/25) among those with incomes of 100 to 400 dollars.

Regarding sexual behaviour, the prevalence of anti-HCV in group II was higher for those who had more numerous sexual partners in the last year, whereas in the case of groups I and III, the prevalence was higher for those with one sexual partner. The absence of a condom during intercourse and the practise of oral/anal intercourse were not statistically significant for anti-HCV positivity in these groups. Although inconsistent condom use presented a significant value of *p* = 0.062 in group II. 

On the other hand, individuals with a sexually transmitted infection had a higher prevalence of anti-HCV in group I (2.9%, 2/69) and group III (80%, 8/10), with a significant *p* value for the coagulopathy group (*p* = 0.031).

Regarding individuals who previously received a blood or plasma transfusion, the highest prevalence of anti-HCV was 15.5% (14/90) in group II and 60% (21/35) in group III, in which results for the coagulopathy group were statistically significant (*p* = 0.004). Moreover, individuals who received blood transfusions prior to 1994 were more inclined to HCV exposure and had an anti-HCV prevalence of 41.2% (7/17) and 64% (16/25), with *p* = 0.004 for the haemodialysis group (Table 4). On the other hand, the use of injectable drugs had a significant *p* value (*p* = 0.064) in group II, albeit not statistically significant.

## 4. Discussion

In the present study, high HBV and HCV prevalence was found in HIV-positive (GI), haemodialysis CRF (GII), and coagulopathies (GIII) populations compared to the general population. We found HBsAg prevalence of 3.9%, 7% and 3.9%, anti-HBc prevalence of 28.6%, 55.9% and 31.4%, and anti-HCV prevalence of 1.3%, 12.6% and 47% in GI, GII and GII, respectively. Pereira et al. [4,5] showed that the HBsAg prevalence ranged from 0.19% to 0.60%, total anti-HBc prevalence ranged from 8, 1% to 10.3%, and anti-HCV prevalence was 1.38% (ranging from 0.68% to 2.60%) in the general population. In Brazil, the prevalence of hepatitis B and C varies according to the geographical region, risk factors, sociodemographic- and population-based characteristics [4,5]. 

HBV prevalence in HIV-infected individuals was similar to previous studies [14,16,17,18]. In the Northeast region of Brazil, Távora et al. [17] found a prevalence of HBV/HIV (3.7%) similar to the present study, despite the different profiles of the health services; we recruited individuals from outpatient HIV/AIDS clinics, while subjects in the previous study were recruited from the infectious disease hospital. Having more than five sexual partners per year and previous sexually transmitted infections correlated with HBV exposure probably due to the shared common transmission routes of both HIV and HBV, particularly through the sexual route [17,24]. As such, this highlights the importance of health education and vaccination programmes for this group to prevent the transmission of HBV. 

In patients with CRF on haemodialysis, the prevalence of HBsAg (7%) and anti-HBc (55.9%) were higher compared to HIV-infected individuals, which could be due to repeated blood exposure during CRF maintenance therapy [25]. The prevalence of both were higher than the prevalence found in the Southeast region (5.5% of HBsAg and 4% of anti-HBc) [19] and in a multicentre Brazilian study (1.4% of HBV prevalence) [26], but lower than the HBsAg prevalence in the South (10%) [27] and Central-West region (46.7%) [28]. These studies showed variable HBV prevalence rates in CRF population that may have been influenced by differences in organizational structure or the work process at haemodialysis centres, which includes the frequency of hygiene and sterilization of equipment in the dialysis room, reuse of capillaries and distribution of patients according to the number of health professionals [8,27,29,30]. 

Patients with CRF on haemodialysis who were older and received 100 to 400 dollars in monthly family income had high anti-HBc prevalence. Older individuals could undergo longer haemodialysis periods, which increases the risk of HBV exposure [31]. In addition, these individuals had difficulty to maintain employment due to the length of time spent on haemodialysis and their clinical condition; these subjects typically received social security benefits (sickness benefit) as a source of income [31,32]. 

Among the coagulopathy group, the prevalence of HBsAg and total anti-HBc was 3.9% and 31.4%, respectively. These results were higher than those found in the South region of Brazil (HBsAg prevalence of 2.9% and prevalence of total anti-HBc of 28.5%) [33,34]. These differences may have been due to the classification of haemophilia and VWD that lead to frequent blood transfusion and a higher risk of exposure to HBV [12,22,23]. In this group, age, history of sexually transmitted disease and blood or plasma transfusion, were associated with a high risk of HBV infection (*p* < 0.05), which was similar to previous observations [35,36,37].

Regarding HCV infection, low anti-HCV prevalence (1.3%) was found in HIV-infected individuals compared to studies conducted in the Northeast region (5.4%) [17] and Southeast region of Brazil (4.6% and 6.4%) [14,16]; this was probably due to the small number of intravenous drug users enrolled in the present study. In CRF patients, the prevalence of anti-HCV was 12.6% which is lower than that found in studies conducted in the Southeast region (14.8%) [9], Northeast region (52%) [38] and South region of Brazil (13.57%) [39], which could have been the result of different standards of good practice for dialysis services [25,29,39]. In this group, receiving a blood transfusion before 1994 was associated with HCV infection (*p* = 0.004), as anti-HCV screening in Brazil was initiated in blood banks in 1993 [40]. 

In coagulopathy patients, anti-HCV prevalence was 47% which is higher than that found in the Southeast region (39.4%) [34], Northeast region (42.2%) [41] and South region of the country (34.16%) [34] and could have been the result of different types of coagulopathy included in each study. The anti-HCV prevalence was 34.9%, 29.7% and 12% in individuals affected by haemophilia A, haemophilia B and VWD, respectively [23]. In this group, a history of sexually transmitted disease and transfusion of blood and/or plasma were associated with high risk of HCV transmission (*p* < 0.05), which reinforced the view that coagulopathy patients who received multiple blood transfusions or blood products [39] prior to 1994 were most affected by HCV due to the lack of anti-HCV screening in blood banks before that date [35,36].

## 5. Conclusions

This study showed a high prevalence of HBV and HCV among the specific groups evaluated compared to the general population. Factors such as age, income, number of sexual partners, history of sexually transmitted disease, blood or plasma transfusion and transfusion before 1994 were associated with the higher prevalence of these infections. These data indicated the necessity of continuous diagnosis, follow-up and prevention of HBV and HCV infections in these groups to prevent clinical complications and the spread of these infections. 

## Figures and Tables

**Table 1:** Sociodemographic characteristics of the sample.

Variable		HIV Positive (*n* = 154)	CRF α Haemodialysis (*n* = 143)	Coagulopathy (*n* = 51)
		*N* (%)	*N* (%)	*N* (%)
**Gender**	Male	131 (85.0)	98 (68.5)	50 (98.0)
	Female	23 (15.0)	45 (31.5)	1 (2.0)
**Age group (years)**	18–25	23 (14.9)	1 (0.7)	15 (29.4)
	26–35	58 (37.7)	24 (16.8)	19 (37.3)
	36–45	46 (29.9)	27 (18.9)	12 (23.5)
	46–55	19 (12.3)	34 (23.8)	4 (7.8)
	56–65	7 (4.5)	36 (25.2)	0 (0.0)
	≥66	1 (0.6)	21 (14.7)	0 (0.0)
	Unknown	0 (0.0)	0 (0.0)	1 (2.0)
**Marital status**	Married/stable union	44 (28.6)	72 (50.3)	17 (33.3)
	Single	109 (70.8)	71 (49.7)	34 (66.7)
	Unknown	1 (0.6)	0 (0.0)	0 (0.0)
**Education**	Illiterate	2 (1.3)	11 (7.7)	2 (3.9)
	1 to 8 years	50 (32.5)	66 (46.1)	18 (35.3)
	>8 years	102 (66.2)	66 (46.1)	31 (60.8)
**Monthly family per capita income**	zero	8 (5.2)	4 (2.8)	4 (7.8)
	<100 dollars	16 (10.4)	22 (15.4)	13 (25.5)
	100 to 400 dollars	75 (48.7)	82 (57.3)	25 (49.0)
	>400 dollars	55 (35.7)	35 (24.5)	9 (17.6)

α CRF = chronic renal failure.

**Table 2:** HBV and HCV markers in population studied.

Variable	HIV-Positive (*n* = 154)	CRF * Haemodialysis (*n* = 143)	Coagulopathy (*n* = 51)
	*N* (%)	*N* (%)	*N* (%)
**Marker of Exposure to HBV**			
HBsAg reactive	6 (3.9%)	10 (7.0%)	2 (3.9%)
Total anti-HBc reactive	44 (28.6%)	80 (55.9%)	16 (31.4%)
Anti-HBs reactive	115 (74.7%)	103 (72.0%)	37 (72.5%)
Anti-HBc reactive/Anti-HBs reactive	33 (21.4%)	55 (38.5%)	15 (29.4%)
**Marker of Exposure to HCV**			
Anti-HCV reactive	2 (1.3%)	18 (12.6%)	24 (47.0%)

* CRF = chronic renal failure.

**Table 3:** Prevalence of anti-HBc antibody in population groups with a high risk for HBV infection.

Variable	HIV Reactive (*n* = 154)	*p*-Value	CRF (*n* = 143)	*p*-Value	Coagulopathy (*n* = 51)	*p*-Value
	Positive/Tested (% Positive)		Positive/Tested (% Positive)		Positive/Tested (% Positive)	
Gender *						
Male	41/131 (31.3)	0.084	55/98 (56.1)	1	16/50 (32.0)	1
Female	3/23 (13.0)		25/45 (55.5)		0/1 (0.0)	
Age Group (years) **						
18–25	5/22 (22.7)	0.678	0/1 (0.0)	0.013	1/15 (6.7)	0.025
26–35	15/58 (25.9)		11/24 (45.8)		7/19 (36.8)	
36– 45	17/46 (36.9)		14/27 (51.8)		6/12 (50.0)	
46–55	5/19 (26.3)		18/34 (52.9)		2/4 (50.0)	
56–65	2/7 (28.6)		25/36 (69.4)		0/0 (0.0)	
≥66	0/1 (0.0)		12/21 (57.1)		0/0 (0.0)	
Marital Status *						
Married/stable union	12/44 (27.3)	1	39/72 (54.2)	0.737	6/17 (35.3)	0.753
Single	32/110 (29.1)		41/71 (57.8)		10/34 (29.4)	
Education **						
Illiterate	0/2 (0.0)	0.891	6/11 (54.5)	0.406	1/2 (50.0)	0.570
1 to 8 years	11/35 (31.4)		28/56 (50.0)		1/11 (9.1)	
>8 years	33/117 (28.2)		46/76 (60.5)		13/37 (35.1)	
Monthly Family per Capita Income **						
0	4/8 (50.0)	0.837	0/1 (0.0)	0.059	3/4 (75.0)	0.121
<100 dollars	3/16 (18.7)		10/21 (47.6)		4/12 (33.3)	
100 to 400 dollars	18/75 (24.0)		43/82 (52.4)		6/25 (24.0)	
>400 dollars	19/55 (34.5)		25/36 (69.4)		3/10 (30.0)	
Sexual Orientation (Males) *						
MSM	30/87 (34.5)	0.312	2/4 (50.0)	1	0/0 (0.0)	0.202
Non-MSM	10/40 (25.0)		52/93 (55.9)		16/49 (32.6)	
Sexual Partner—Last Year **						
1	11/66 (16.7)	0.003	40/72 (55.5)	0.267	9/20 (45.0)	0.543
<5	20/49 (40.8)		11/22 (50.0)		3/16 (18.7)	
6 to 10	7/15 (46.7)		2/2 (100.0)		0/4 (0.0)	
>10	3/6 (50.0)		3/3 (100.0)		1/3 (33.3)	
Unknown	3/18 (16.7)		24/44 (54.5)		3/8 (37.5)	
Condom Use **						
Always	29/112 (25.9)	0.422	21/35 (60.0)	0.285	6/17 (35.3)	0.704
Sometimes	12/29 (41.4)		14/21 (66.7)		6/20 (30.0)	
Never	2/8 (25.0)		42/81 (51.8)		3/9 (33.3)	
Unknown	1/5 (20.0)		3/6 (50.0)		1/5 (20.0)	
**ARV Treatment**						
Yes	6/123 (4.9)	0.600				
No	0/31 (0.0)		-		-	
Unknown	12/194 (6.2)					
**Sexually Transmitted Infection**						
Yes	29/69 (42.0)	0.001	23/39 (58.9)	0.707	8/10 (80.0)	0.000
No	15/84 (17.8)		56/102 (54.9)		8/40 (20.0)	
Unknown						
**Oral Intercourse**						
Yes	37/115 (32.2)	0.092	31/54 (57.4)	1	7/25 (28.0)	0.537
No	6/37 (16.2)		34/60 (56.7)		8/21 (38.1)	
Unknown	1/2 (50.0)		15/29 (51.7)		1/5 (20.0)	
**Anal Intercourse**						
Yes	35/114 (30.7)	0.302	26/50 (52.0)	0.459	6/19 (31.6)	1
No	8/38 (21.0)		43/72 (59.7)		9/27 (33.3)	
Unknown	1/2 (50.0)		11/21 (52.4)		1/5 (20.0)	
**Tattoo**						
Yes	12/42 (28.6)	1	5/13 (38.5)	0.243	2/6 (33.3)	1
No	32/112 (28.6)		75/130 (57.7)		14/44 (31.8)	
Unknown	0		0		0/1 (0.0)	
**Piercing**						
Yes	4/15 (26.7)	1	1/2 (50.0)	1	1/2 (50.0)	1
No	40/139 (28.8)		79/141 (56.0)		15/47 (31.9)	
Unknown	0		0		0/2 (0.0)	
**Transfusion of Blood/Plasma**						
Yes	5/16 (31.2)	0.779	51/90 (56.7)	0.861	15/35 (42.8)	0.018
No	39/136 (28.7)		28/51 (54.9)		1/15 (6.7)	
Unknown	0/2 (0.0)		1/2 (50.0)		0/1 (0.0)	
**Transfusion before 1994**						
Yes	3/8 (37.5)	0.569	11/15 (73.3)	0.161	13/25 (52.0)	0.076
No	1/8 (12.5)		39/75 (52.0)		2/11 (18.2)	
Unknown	40/138 (29.0)		30/53 (56.6)		1/15 (6.7)	
**Attend Manicure**						
Yes	24/94 (25.5)	0.460	45/80 (56.2)	1	6/20 (30.0)	1
No	19/59 (32.2)		34/62 (54.8)		10/30 (33.3)	
Unknown	1/1 (100.0)		1/1 (100.0)		0/1 (0.0)	
**Injecting/Inhaled Drug Use**						
Yes	8/34 (23.5)	0.525	3/8 (37.5)	0.466	1/6 (16.7)	0.649
No	36/120 (30.0)		76/134 (56.7)		15/43 (34.9)	
Unknown	0		1/1 (100.0)		0/2 (0.0)	

CRF = chronic renal failure; * Fisher’s exact test; ** chi-square test for trend.

**Table 4:** Prevalence of anti-HCV antibody in population groups with a high risk for HCV infection.

Variable	HIV Reactive (*n* = 154)	*p*-Value	CRF (*n* = 143)	*p*-Value	Coagulopathy (*n* = 51)	*p*-Value
	Positive/Tested (% Positive)		Positive/Tested (% Positive)		Positive/Tested (% Positive)	
Gender *						
Male	1/131 (3.2)	0.277	15/98 (15.3)	0.182	24/50 (48.0)	1
Female	1/23 (4.3)		3/45 (6.7)		0/1 (0.0)	
**Age Group (Years) ^α^**						
18–25	0/22 (0.0)	0.367	0/1 (0.0)	0.394	2/15 (13.3)	0.474
26–35	0/58 (0.0)		1/24 (4.1)		9/19 (47.4)	
36–45	1/46 (2.1)		5/27 (18.5)		11/12 (91.6)	
46–55	1/19 (5.2)		2/34 (5.9)		1/4 (25.0)	
56–65	0/7 (0.0)		8/36 (22.2)		0/0 (0.0)	
≥66	0/1 (0.0)		2/21 (9.5)		0/0 (0.0)	
Marital Status *						
Married/stable union	1/44 (2.3)	0.491	9/72 (12.5)	1	11/17 (64.7)	0.135
Single	1/110 (0.9)		9/71 (12.7)		13/34 (38.2)	
**Education ^α^**						
Illiterate	0/2 (0.0)	0.128	1/11 (9.0)	0.144	1/2 (50.0)	0.404
1 to 8 years	1/35 (2.8)		11/56 (19.6)		6/11 (54.5)	
>8 years	1/117 (0.8)		6/76 (7.9)		16/37 (43.2)	
**Monthly Family Per Capita Income ^α^**						
Zero	0/8 (0.0)	0.481	0/1 (0.0)	0.412	4/4 (100.0)	0.210
<100 dollars	1/16 (6.4)		3/21 (14.3)		3/12 (25.0)	
100 to 400 dollars	0/75 (0.0)		11/82 (13.4)		14/25 (56.0)	
>400 dollars	1/55 (1.8)		3/36 (8.3)		3/10 (30.0)	
Sexual Orientation (Males) *						
MSM	1/87 (1.1)	1	1/4 (25.0)	0.495	0/0 (0.0)	1
Non-MSM	0/40 (0.0)		14/93 (15.0)		23/49 (46.9)	
**Sexual Partner—Last Year ^α^**						
1	1/66 (1.5)	0.331	6/72 (8.3)	0.214	13/21 (61.9)	0.214
<5	0/49 (0.0)		5/22 (22.7)		6/16 (37.5)	
6 to 10	0/15 (0.0)		2/2 (100.0)		1/4 (25.0)	
>10	0/6 (0.0)		1/3 (33.3)		1/3 (33.3)	
Unknown	1/18 (5.6)		4/44 (9.1)		3/7 (42.9)	
**Condom Use ^α^**						
Always	2/112 (1.8)	0.165	7/35 (20.0)	0.062	9/18 (50.0)	0.938
Sometimes	0/29 (0.0)		3/22 (13.6)		8/19 (42.1)	
Never	0/8 (0.0)		7/81 (8.6)		5/9 (55.5)	
Unknown	0/5 (0.0)		1/5 (20.0)		2/5 (40.0)	
**Sexually Transmitted Infection**						
Yes	2/69 (2.9)	0.201	4/39 (10.2)	0.779	8/10 (80.0)	0.031
No	0/84 (0.0)		14/102 (13.7)		15/40 (37.5)	
Unknown	1		2		1	
**Oral Intercourse**						
Yes	2/115 (1.7)	1	5/54 (9.2)	0.275	9/25 (36.0)	0.138
No	0/37 (0.0)		10/60 (16.6)		13/21 (61.9)	
Unknown	0/2 (0.0)		3/27 (11.1)		2/5 (40.0)	
**Anal Intercourse**						
Yes	1/114 (0.8)	0.438	7/50 (14.0)	0.780	8/19 (42.1)	0.561
No	1/38 (2.6)		8/72 (11.1)		14/27 (51.8)	
Unknown	0/2 (0.0)		1/21 (4.8)		2/5 (40.0)	
**Tattoo**						
Yes	1/42 (2.4)	0.472	0/13 (0)	0.372	3/6 (50.0)	1
No	1/112 (0.9)		18/130		13/21 (61.9)	
Unknown	0		0		1/1 (100.0)	
**Piercing**						
Yes	1/15 (6.6)	0.186	0/2 (0.0)	1	2/2 (100.00)	0.215
No	1/139 (0.7)		18/141 (12.7)		21/47 (44.7)	
Unknown	0		0		1/2 (50.0)	
**Transfusion of Blood/Plasma**						
Yes	0/16 (0.0)	1	14/90 (15.5)	0.110	21/35 (60)	0.004
No	2/136 (1.5)		3/51 (5.9)		2/15 (13.3)	
Unknown	0/2 (0.0)		1/2 (50.0)		1/1 (100.0)	
**Transfusion before 1994**						
Yes	0/8 (0.0)	1	7/17 (41.2)	0.004	16/25 (64.0)	0.464
No	0/8 (0.0)		7/73 (9.6)		5/11 (45.4)	
Unknown	2/138 (1.4)		4/53 (7.5)		3/15 (20.0)	
**Attend Manicure**						
Yes	2/94 (2.1)	0.523	7/80 (8.7)	0.201	9/20 (45.0)	1
No	0/59 (0.0)		10/62 (16.1)		14/30 (46.6)	
Unknown						
**Injecting/Inhaled Drug Use**						
Yes	1/34 (2.9)	0.393	3/8 (37.5)	0.064	3/6 (50.0)	1
No	1/120 (0.8)		15/134 (11.2)		20/43 (46.5)	
Unknown	0/0 (0.0)		0/1 (0.0)		1/2 (50.0)	

CRF = chronic renal failure; ^α^ Chi-Squared for trend; * Fisher’s exact test.
